# Moxibustion for Chronic Fatigue Syndrome: A Systematic Review and Meta-Analysis

**DOI:** 10.1155/2021/6418217

**Published:** 2021-11-11

**Authors:** Jianyu You, Jing Ye, Haiyan Li, Wenguo Ye, Ensi Hong

**Affiliations:** ^1^Jiangxi University of Chinese Medicine, Nanchang, China; ^2^The Affiliated Hospital of Jiangxi University of Chinese Medicine, Nanchang, China

## Abstract

**Objective:**

This review aimed at systematically evaluating the efficacy and safety of moxibustion for chronic fatigue syndrome (CFS).

**Methods:**

Relevant trials were searched in seven digital databases up to January 2021. After literature screening, data extraction, and literature quality evaluation, the included studies were meta-analyzed using RevMan 5.4 software. The evidence level was assessed using the Grading of Recommendations, Assessment, Development, and Evaluation (GRADE).

**Results:**

Fifteen studies involving 1030 CFS participants were included. Meta-analyses showed a favorable effect of moxibustion on the total effective rate compared with acupuncture (OR = 4.58, 95%CI = [2.85, 7.35], *P* < 0.00001) and drugs (OR = 6.36, 95%CI = [3.48, 11.59], *P* < 0.00001). Moxibustion also appeared to significantly reduce fatigue severity measured by fatigue scale-14 (FS-14) (WMD = −2.20, 95% CI = [−3.16, −1.24], *P* < 0.00001) and fatigue assessment instrument (FAI) (WMD = −16.36, 95% CI = [−26.58, −6.14], *P*=0.002) compared with the control group. In addition, among the 15 included studies, only two studies reported adverse events related to moxibustion, and the symptoms were relatively mild. The quality of evidence based on the 15 included trials was assessed as moderate to very low.

**Conclusions:**

Based on limited evidence, moxibustion might be an effective and safe complementary therapy for CFS, which can be recommended to manage CFS. Because of the limited level of evidence in this review, further high-quality trials are still needed to confirm these findings.

## 1. Introduction

Myalgic encephalomyelitis/chronic fatigue syndrome (ME/CFS) is a disabling clinical condition characterized by unexplained and persistent postexertional fatigue accompanied by a variety of symptoms related to cognitive, autonomous dysfunction, and immunological, including profound fatigue, orthostatic intolerance, unrefreshing sleep, and cognitive deficits [1]. It is estimated that the global average prevalence of CFS in adults is 0.65%. When defined by the most commonly used cases, this proportion rises to 0.89% [2, 3]. Although CFS is not life-threatening, it seriously affects the patient's quality of life and causes a tremendous socioeconomic burden [4, 5]. According to the latest report from the Institute of Medicine of the United States, approximately 836,000 to 2.5 million Americans suffer from CFS [6], which incurs annual costs ranging from US$1.8 to 24 billion per year [7].

To date, the pathogenesis of CFS is still unclear, and the Institute of Medicine (IOM) defines CFS as a complex multisystem neurological disease [8]. It is generally believed that the pathogenesis of CFS may be related to various factors, including brain structure and function, immune function, neuroendocrine response, viral infections, sleep architecture, and biopsychosocial models [9, 10]. Since the etiology of CFS is unclear, the treatment of CFS mainly focuses on relieving symptoms [11]. However, there are currently no specific Food and Drug Administration (FDA)-approved drugs for the treatment of CFS [12]. In addition, cognitive behavioral therapy (CBT) and graded exercise therapy (GET) are considered promising therapies for managing CFS [13]. However, recent studies have shown that the regulatory effects of CBT and GET are limited, and their effectiveness is still controversial [14–16]. Due to the limited overall therapeutic effects of CFS, some researchers have begun to turn their attention to complementary and alternative medicine (CAM).

Among various CAM therapies, moxibustion has been widely used in the management of various health conditions in China and has received widespread attention. As an ancient external treatment with a history of 2500 years, it involves using the heat of burning moxibustion to stimulate acupoints or specific surficial regions to relieve the symptoms of patients. According to the description in ancient Chinese literature, the therapeutic effect of moxibustion is related to improving the “weakness” symptoms of patients and preventing human diseases [17]. This makes moxibustion used as a complementary therapy for many diseases, including CFS.

Although the benefits of moxibustion for CFS have been widely reported [18], and some related systematic reviews have been conducted before, these systematic reviews have some limitations [19, 20]. None of them evaluated the efficacy of using a single moxibustion. Therefore, we conducted a new systematic review to evaluate the efficacy of moxibustion alone in the treatment of CFS.

## 2. Methods

### 2.1. Data Source and Search Strategy

Seven online databases were searched from their inception to January 2021: PubMed, the Cochrane Library, EMBASE, CBM, CNKI, VIP, and Wanfang database. The search method used a combination of MeSH terms and free words, and the search terms were composed of intervention methods (moxibustion) and disease names (chronic fatigue symptoms). PubMed retrieval strategies are shown in Additional file 1. References listed in the included trials were also screened to identify potential trials.

### 2.2. Eligibility Criteria

Inclusion criteria were defined as follows: (1) the study was a randomized controlled trial (RCT); (2) subjects met the CFS diagnostic criteria established by the Centers for Disease Control and Prevention (for example, CDC 1994); (3) the intervention methods of the experimental group only included moxibustion, and there was no restriction on the type of moxibustion therapy; (4) the control group included active treatments (e.g., drugs, acupuncture, CBT, GET) or no treatment, and the drugs here do not contain Chinese herbal medicine; and (5) outcome indicators: (i) clinical efficacy (The clinical efficacy is mainly based on the standards established in the “Foreign Medical Sciences-Chinese Medicine fascicles,” which is defined as effective when the main clinical symptoms and concurrent symptoms are improved by more than 1/3 or 30%; otherwise, it is considered invalid [21]. In addition, other clinical efficacy evaluation criteria with comparable definitions were also considered), (ii) fatigue severity (measured by validated scales such as fatigue scale-14 (FS-14) and fatigue assessment instrument (FAI)), and (iii) adverse events (AEs). Exclusion criteria were defined as follows: (1) duplicate data; (2) non-RCT; (3) lack of definitive diagnostic criteria; (4) unusable data; and (5) the experimental group did not use moxibustion alone or the control group included moxibustion or Chinese herbal medicine.

### 2.3. Data Extraction

Two investigators independently browsed all the titles, abstracts, and full texts to screen eligible trials. Disagreements were resolved through discussion. Collected data included the following: study author, article publication date and location, and basic information of included trials (sample size, gender, age, intervention, and outcomes).

### 2.4. Study Quality Assessment

The quality assessment was performed independently by two investigators using the Cochrane risk of bias (ROB) tool [22], which included six items: the implementation of randomization, allocation concealment, blinding, the integrity of data, outcome reporting, and other biases. Each domain was graded three levels as low, high, or unclear ROB. Any inconsistencies were resolved by consulting a third investigator.

### 2.5. Data Synthesis and Analysis

RevMan 5.4 software was used for statistical analysis. The odds ratio (OR) with 95% confidence intervals (CI) were calculated for categorical data (Clinical efficacy), and weighted mean difference (WMD) with 95% CIs were calculated for continuous variables (FS-14 and FAI). Heterogeneity between trials was assessed by the *χ*^2^ test and *I*^*2*^ test. If found homogenous (*I*^*2*^ ≤ 50% and *P* ≥ 0.10), then the fixed effect model was used; otherwise, the random-effects model was applied, and the sources of heterogeneity were explored using subgroup analysis or sensitivity analysis. We conducted subgroup analysis based on the differences in the control group and the type of moxibustion. Egger's test was used to analyze potential publication bias (more than 10 studies). In addition, the certainty of evidence was evaluated according to the GRADE system and was divided for each outcome index into four categories, including high, medium, low, and very low.

## 3. Results

### 3.1. Literature Search

**Figure 1:**
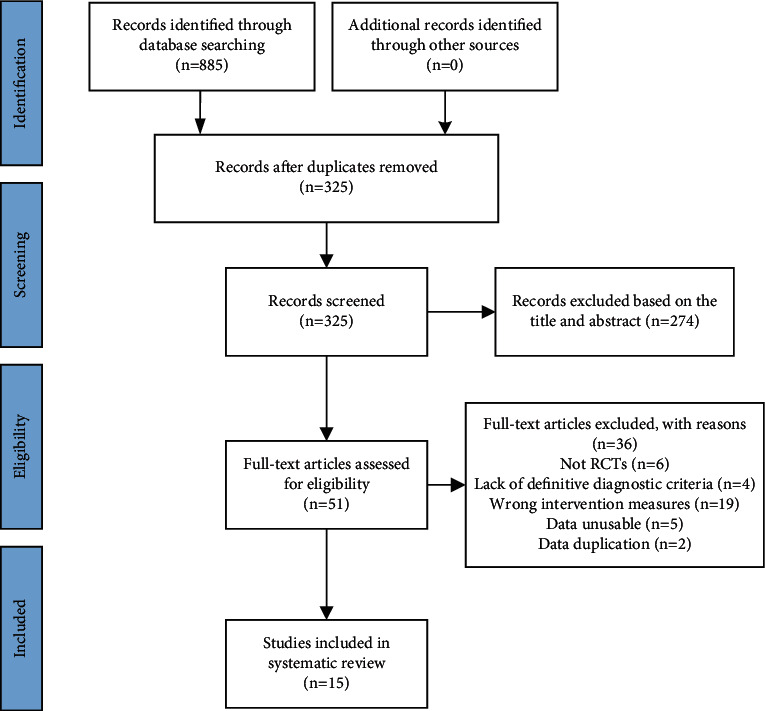
Flowchart of the study selection process.

885 publications were retrieved from initial search. After deleting duplicates and reading the title, abstract, and full texts, finally, 15 trials [23–37] were included. The PRISMA flowchart of the literature search is shown in Figure 1.

### 3.2. Study Characteristics

Among the 15 RCTs, all trials were conducted in different provinces of China and the publication year was between 2007 and 2020. This study involved a total of 1030 CFS patients (520 in the moxibustion group, 510 in the control group). There were 10 trials [25, 29–37] that compared single moxibustion with acupuncture, and the remaining 5 trials [23, 24, 26–28] compared single moxibustion with drugs.  Table 1 shows the detailed information of all included RCTs.

### 3.3. Risk of Bias

**Figure 2:**
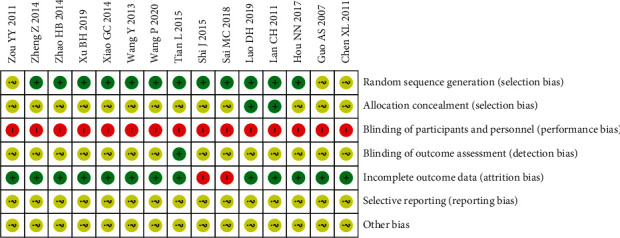
Potential risk of bias of each included study.

In all 15 RCTs, twelve trials [25, 27–37] clearly reported the implementation method of randomization, while in the other three trials [23, 24, 26], the specific details of randomization were not mentioned. Only two trials [25, 35] mention the details of using allocation concealment. Due to the particularity of moxibustion operation, blinding the patient is not feasible. Only one study [32] mentioned the details of blinding, which implemented blinding in the outcome assessment process. Two trials [31, 34] mentioned dropouts without detail information of handling. No reporting bias was found among the included 15 RCTs. Since all RCTs were not registered in advance, other biases were classified as unclear. The Cochrane ROB assessment is shown in Figure 2.

### 3.4. Clinical Efficacy

**Figure 3:**
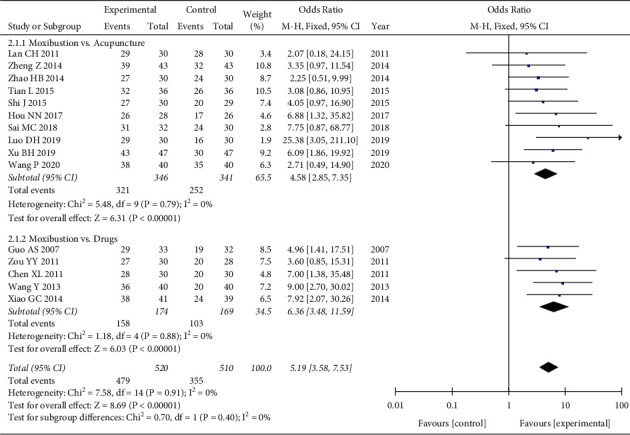
Forest plot of moxibustion on the total effective rate.

All trials reported the effective rate of moxibustion in relieving CFS. No heterogeneity was found (*P*=0.91, *I*^*2*^ = 0%), and the fixed-effects model showed that moxibustion was better than the control group in improving the effective rate (OR = 5.19, 95%CI = [3.58, 7.53], *P* < 0.00001). The results of subgroup analysis also showed that moxibustion was better than acupuncture (OR = 4.58, 95%CI = [2.85, 7.35], *P* < 0.00001) and drugs (OR = 6.36, 95%CI = [3.48, 11.59], *P* < 0.00001) (Figure 3).

### 3.5. FS-14

**Figure 4:**
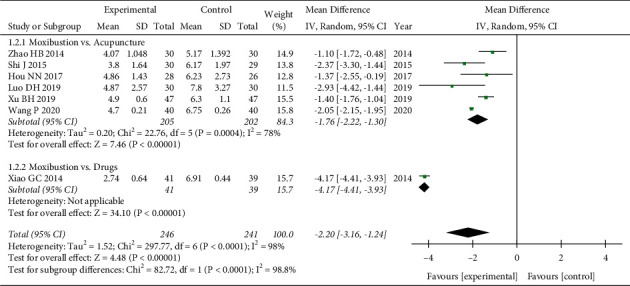
Forest plot of different control groups for FS-14.

**Figure 5:**
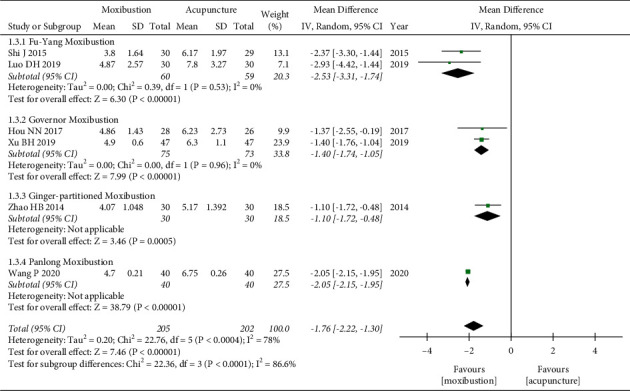
Forest plot of different moxibustion modalities for FS-14.

Seven trials [28, 29, 31, 33, 35–37] evaluated fatigue severity by using FS-14. Analysis of data showed obvious heterogeneity (*P* < 0.00001, *I*^*2*^ = 98%), and the random-effects model showed that moxibustion could further relieve SF-14 compared with the control group (WMD = −2.20, 95% CI = [−3.16, −1.24], *P* < 0.00001). Subgroup analysis based on the type of control group also showed that moxibustion was better than acupuncture (WMD = −1.76, 95%CI = [−2.22, −1.30], *P* < 0.00001) and drugs (WMD = −4.17, 95%CI = [−4.41, −3.93], *P* < 0.00001). However, the heterogeneity of the meta-analysis of moxibustion versus acupuncture was still high. We conducted a subgroup analysis based on the type of moxibustion, and the results showed that the two subgroups Fu-Yang moxibustion (*P*=0.53, *I*^*2*^ = 0%) and governor moxibustion (*P*=0.96, *I*^*2*^ = 0%) did not find significant heterogeneity, and the four different types of moxibustion (Fu-Yang moxibustion, governor moxibustion, ginger-partitioned moxibustion, and Panlong moxibustion) can further relieve SF-14 compared with the acupuncture group, and Fu-Yang moxibustion (WMD = −2.53, 95%CI = [−3.31, −1.74], *P* < 0.00001) seems to be better. The test of subgroup differences showed that the different control types (*P* < 0.00001) and moxibustion types (*P* < 0.00001) may cause heterogeneity (Figures 4 and 5).

### 3.6. FAI

**Figure 6:**
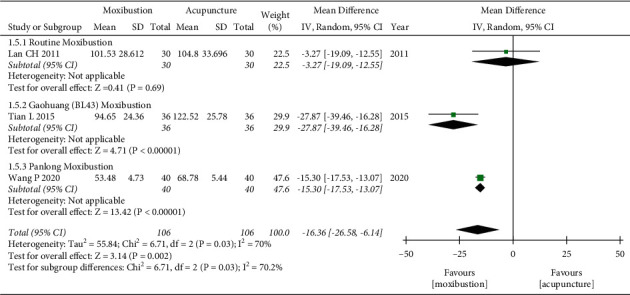
Forest plot of moxibustion on FAI.

Three trials [25, 32, 37] evaluated fatigue severity by using FAI scores. Since heterogeneity was found between the three RCTs (*P*=0.03, *I*^*2*^ = 70%), a random-effects model was used. Our pooled results showed that moxibustion could further improve the FAI score compared with acupuncture (WMD = −16.36, 95% CI = [−26.58, −6.14], *P*=0.002). Subgroup analysis based on the type of moxibustion also showed that Gaohuang (BL43) moxibustion and Panlong moxibustion were better than acupuncture. However, there was no statistically significant difference between routine moxibustion and acupuncture based on one study [25]. The test of subgroup differences indicated that the moxibustion types might lead to heterogeneity (*P*=0.03) (Figure 6).

### 3.7. Safety Assessment

Eight trials [25, 27, 28, 30–33, 35] reported details of adverse events (AEs), and six [27, 28, 30–33] of them reported no adverse events. Two trials [25, 35] reported moxibustion-related AEs, and both reported 1 case of mild scald. Two trials [25, 35] reported acupuncture-related AEs, one [25] reported 3 cases of dizziness during acupuncture, and one [35] reported 2 cases of local hematoma at the acupuncture site. The symptoms of the above-mentioned adverse events were relatively mild, and none of them affected the patient's follow-up treatment.

### 3.8. Heterogeneity and Sensitivity Analysis

There was obvious heterogeneity in the comparison of moxibustion versus acupuncture on the FS-14 (*I*^*2*^ = 78%) and FAI score (*I*^*2*^ = 70%). We performed subgroup analysis based on the type of moxibustion, and the subgroup difference test showed that different modalities of moxibustion treatment may be the cause of the heterogeneity. Due to the small number of included studies for these two outcome indicators (less than 10 studies), we conducted sensitivity analysis by the conversion effect model. Sensitivity analysis indicated that the results of the meta-analysis were stable.

### 3.9. Publication Bias

**Figure 7:**
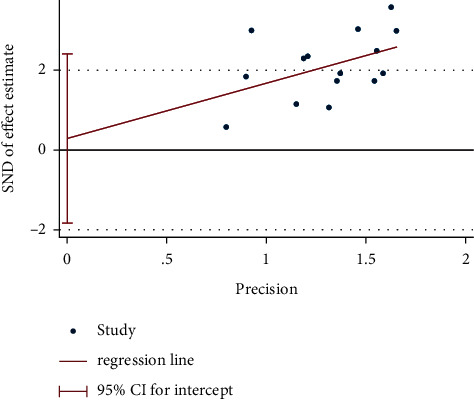
Egger's test plot of moxibustion on the total effective rate.

We used Egger's test to analyze the publication bias of the total effective rate (more than 10 studies), and the results showed that the publication bias was not significant (*P*=0.772) (Figure 7).

### 3.10. Certainty of Evidence

The results of the GRADE analysis are shown in Table 2. In general, in addition to the certainty of evidence for the clinical efficacy of moxibustion versus acupuncture, which was rated as “moderate,” the other outcome indicators were rated as “low” or “very low.” The main reasons leading to the decline in the certainty of the evidence for the outcome indicators include the methodological quality of most of the included studies was not high and the sample size was small, and the heterogeneity of some outcome indicators is obvious.

## 4. Discussion

To the best of our knowledge, this is the first meta-analysis to evaluate the efficacy of a single moxibustion treatment for CFS. In our current study, we included 15 RCTs that compared moxibustion with acupuncture (10 RCTs) and drugs (5 RCTs). Our pooled analysis indicated that moxibustion was significantly better than acupuncture in relieving fatigue symptoms (FS-14 and FAI) and improving clinical efficacy (*P* < 0.05). In addition, compared with drugs, moxibustion has an advantage in improving the clinical efficacy and reducing the FS-14 score (*P* < 0.05). Although the clinical efficacy in this study was evaluated according to Chinese standards, this criterion involves the comprehensive evaluation of the main symptoms and accompanying symptoms of CFS patients. Therefore, we believe that the analysis conclusions of the clinical efficacy of this study are reliable. In addition, fatigue is one of the most important clinical symptoms of CFS. Both FS-14 and FAI clinically are internationally recognized measurement tools for evaluating fatigue symptoms, which can truly reflect the severity of fatigue [38, 39]. Therefore, it is also credible to use FS-14 and FAI as outcome indicators for evaluating the efficacy of CFS. In terms of safety assessment, six studies reported no adverse events. Two studies reported adverse events of moxibustion, such as scald during moxibustion, but with mild symptoms. These adverse events can be effectively minimized by standardizing the operation steps of moxibustion [40, 41]. Therefore, based on the findings of this study, we suggest that moxibustion might be an effective and safe complementary therapy for CFS. However, it cannot be ignored that the level of currently available evidence has been evaluated by the GRADE system as “moderate,” “low,” or “very low.” This greatly weakens the reliability and impact of the evidence, which suggests that the interpretation of these positive results should be cautious. Therefore, the efficacy of moxibustion for CFS needs to be further explored.

Moxibustion is a traditional Chinese medicine method widely used in East Asia. The theoretical basis of moxibustion is the same as that of acupuncture, and they are guided by the theory of meridians and acupoints. However, since the application of moxibustion in clinics is not as extensive as acupuncture, the popularity and application of moxibustion in Western countries are not as good as acupuncture. In fact, like acupuncture, moxibustion also has the advantages of easy operation, safety, and economy [40, 42–44]. TCM theory believes that moxibustion can regulate the balance of Qi and blood in the body by warming the meridians, unblocking the collaterals, and promoting the movement of Qi and blood, thereby restoring the body's Yang Qi vitality. This makes moxibustion widely used to treat diseases with “weakness” symptoms, including CFS [32, 45]. In addition, modern research provides laboratory-based evidence that moxibustion can effectively regulate the behavior, immune function, and hypothalamic-pituitary-adrenal axis hormone levels of CFS model rats [46–48], thereby alleviating fatigue symptoms.

There were some limitations in this study. First, the methodological quality of most included trials in the Cochrane ROB assessment was not satisfactory. Only two trials implemented allocation concealment, and only one RCT reported blind details about the result evaluation. Second, all 15 included trials were from Chinese databases, which may cause language bias. Third, the sample size of most trials was small. Finally, the heterogeneity of the meta-analysis results (FS-14 and FAI) was high, which may be related to the moxibustion treatment plan of the experimental group (FS-14 and FAI) and the type of control group (FS-14). All of the above factors may limit the accuracy of the conclusions of this study.

## 5. Conclusion

Based on limited evidence, our research results show that moxibustion might be an effective and safe complementary therapy for CFS, especially in improving clinical efficacy and relieving fatigue symptoms. Due to the limited level of evidence, further high-quality RCTs are still needed to confirm the benefits of moxibustion for CFS.

## Figures and Tables

**Table 1:** Characteristics of included studies.

Study	Study location	Sample size (male/female)	Mean age (SD)	Interventions group	Control group	Treatment period	Outcomes
Guo et al. (2007) [23]	Jiangsu, China	T: 14/19 C: 12/20	T: 36.01 + 6.74 C: 35.12 + 7.30	Moxibustion	Drug (fluoxetine)	T: once a day for 30 days, 30 min C: once a day for 30 days	CE
Chen et al. (2011) [24]	Guangdong, China	T: 16/14 C: 17/13	T: 35.12 ± 4.17 C: 35.91 ± 3.25	Moxibustion	Drug (vitamin C + vitamin E + trivitamins B tablets)	T: once a day for 28 days, 10–20 min C: three times a day for 28 days	CE
Lan (2011) [25]	Guizhou, China	T: 16/14 C: 15/15	T: 30.70 + 8.801 C: 30.80 + 11.370	Moxibustion	Acupuncture	T: once a day, ten days per period, one days' break between two periods, two periods in total, 20 min C: once a day, ten days per period, one days' break between two periods, two periods in total, 20 min	CE, FAI, AEs
Zou (2011) [26]	Jiangxi, China	T: 15/15 C: 13/15	T: 34.67 ± 3.24 C: 35.72 ± 3.76	Moxibustion	Drug (fluoxetine)	T: once a day, eight days per period, three days' break between two periods, three periods in total, 15 min C: once a day for 30 days	CE
Wang et al. (2013) [27]	Anhui, China	T: 12/28 C: 13/27	T: 39 ± 6 C: 38 ± 8	Thunder-fire moxibustion	Drug (fluoxetine)	T: once a day, ten days per period, two days' break between two periods, two periods in total C: once a day for 20 days	CE, AEs
Xiao et al. (2014) [28]	Guizhou, China	T: 20/21 C: 19/20	T: 40.2 ± 8.2 C: 40.6 ± 8.5	Moxibustion	Drug (vitamin B1 + vitamin B6 + oryzanol + paroxetine)	T: once a day, ten days per period, three days' break between two periods, three periods in total, 15 min C: NR	CE, FS-14, AEs
Zhao (2014) [29]	Heilongjiang, China	T: 10/20 C: 9/21	T: 40.80 ± 6.599 C: 41.07 ± 5.783	Ginger-partitioned moxibustion	Acupuncture	T: once a day, five times per week for 4 weeks, 50 min C: once a day, five times per week for 4 weeks, 30 min	CE, FS-14
Zheng et al. (2014) [30]	Guangdong, China	T: 23/20 C: 21/22	T: 43.5 ± 13.2 C: 42.6 ± 12.9	Moxibustion	Acupuncture	T: once a day, seven days per period, three days' break between two periods, three periods in total C: once a day, seven days per period, three days' break between two periods, three periods in total, 30 min	CE, AEs
Shi (2015) [31]	Guangdong, China	T: 8/22 C: 9/20	T: 39.00 ± 12.54 C: 41.62 ± 11.70	Fu-Yang moxibustion	Acupuncture	T: twice a week for six weeks C: once a day, three times per week for six weeks	CE, FS-14, AEs
Tian et al. (2015) [32]	Gansu, China	T: 24/12 C: 16/20	T: 42 ± 9 C: 42 ± 10	Gaohuang (BL43) moxibustion	Acupuncture	T: once a day, ten days per period, two days' break between two periods, three periods in total C: once a day, ten days per period, two days' break between two periods, three periods in total, 30 min	CE, FAI, AEs
Hou et al. (2017) [33]	Shandong, China	T: 16/12 C: 14/12	T: 43.07 ± 9.31 C: 45.62 ± 9.92	Governor moxibustion	Acupuncture	T: twice a month for 3 months, 6 hours C: once a day, four times per week for 3 months, 30 min	CE, FS-14, AEs
Sai (2018) [34]	Shandong, China	T: 17/15 C: 13/17	T: 38.97 ± 6.98 C: 37.73 ± 6.26	Viscera moxibustion	Acupuncture	T: once a week for 8 weeks, 2 hours C: once a day, three times per week for 8 weeks, 30 min	CE
Luo et al. (2019) [35]	Guangdong, China	T: 16/14 C: 15/15	T: 43 ± 4 C: 42 ± 3	Fu-Yang moxibustion	Acupuncture	T: once every two days for 60 days C: once every two days for 60 days, 30 min	CE, FS-14, AEs
Xu et al. (2019) [36]	Henan, China	T: 29/18 C: 27/20	T: 41.5 ± 5.3 C: 42.5 ± 3.6	Governor moxibustion	Acupuncture	T: twice a month for 3 months, 6 hours C: once a day, four times per week for 3 months, 30 min	CE, FS-14
Wang et al. (2020) [37]	Hunan, China	T: 13/27 C: 15/25	T: 43.00 ± 1.03 C: 43.00 ± 1.17	Panlong moxibustion	Acupuncture	T: once a week for 3 weeks, 2 hours C: once a day, five times per week for 3 weeks, 30 min	CE, FS-14, FAI

AEs, adverse events; C, control group; CE, clinical efficacy; FAI, fatigue assessment instrument; FS-14, fatigue scale-14; NR: not reported; T, therapy group.

**Table 2:** GRADE certainty grading evaluation.

Certainty assessment							No. of patients		Effect (95% CI)	Certainty
No. of studies	Design	Risk of bias	Inconsistency	Indirectness	Imprecision	Publication bias	Experimental group	Control group		
Clinical efficacy (moxibustion vs. acupuncture)										
10	Randomized trials	Serious^a^	No serious inconsistency	No serious indirectness	No serious imprecision	None	321/346 (92.8%)	252/341 (73.9%)	OR 4.58 (2.85, 7.35)	⊕⊕⊕○ Moderate
FS-14 (moxibustion vs. acupuncture)										
6	Randomized trials	Serious^a^	Very serious^b^	No serious inconsistency	No serious imprecision	None	205	202	MD −1.76 (−2.22, −1.30)	⊕○○○ Very low
FAI (moxibustion vs. acupuncture)										
3	Randomized trials	Serious^a^	Serious^b^	No serious inconsistency	Serious^c^	None	106	106	MD −16.36 (−26.58, −6.14)	⊕○○○ Very low
Clinical efficacy (moxibustion vs. drugs)										
5	Randomized trials	Serious^a^	No serious inconsistency	No serious indirectness	Serious^c^	None	158/174 (90.8%)	103/169 (60.9%)	OR 6.39 (3.48, 11.59)	⊕⊕○○ Low
FS-14 (moxibustion vs. drugs)										
1	Randomized trials	Serious^a^	No serious inconsistency	No serious inconsistency	Very serious^c^	None	41	39	MD −4.17 (−4.41, −3.93)	⊕○○○ Very low

^a^Risk of bias: most studies had a high risk of bias in methodology. ^b^Inconsistency: considerable heterogeneity. ^c^Imprecision: small sample size.

## Data Availability

This systematic review is a secondary analysis of the published RCTs data. All the study data can be accessed through the original articles listed in the “References” section.

## Supplementary Materials

Supplementary Materials Supplemental materials show the complete search process in PubMed. Our search terms include disease name (chronic fatigue symptoms) and intervention method (moxibustion).
