# Supplementary material for: Moxibustion for Chronic Fatigue Syndrome: A Systematic Review and Meta-Analysis
Source: Evid Based Complement Alternat Med. 2021 Nov 11;2021:6418217. doi: 10.1155/2021/6418217 (PMC8601810; doi:10.1155/2021/6418217)
Supplement: Supplementary Materials — Supplemental materials show the complete search process in PubMed. Our search terms include disease name (chronic fatigue symptoms) and intervention method (moxibustion). [file 6418217.f1.pdf]

Supplementary Material: Search strategy in PubMed database

Number	Search terms
#1	Fatigue syndrome, chronic [MeSH]
#2	Chronic fatigue syndrome* [Title/Abstract]
#3	Myalgic encephalomyelitis [Title/Abstract]
#4	Postviral Fatigue Syndrome* [Title/Abstract]
#5	Infectious Mononucleosis Like Syndrome [Title/Abstract]
#6	Royal Free Disease [Title/Abstract]
#7	Chronic Fatigue [Title/Abstract]
#8	Fatigue Disorder* [Title/Abstract]
#9	Systemic Exertion Intolerance Disease [Title/Abstract]
#10	Fatigue Syndrome* [Title/Abstract]
#11	CFS [Title/Abstract]
#12	ME [Title/Abstract]
#13	#1 OR #2 OR #3 OR #4 OR #5 OR #6 OR #7 OR #8 OR #9 OR #10 OR #11 OR #12
#14	Moxibustion [Mesh]
#15	Moxibustion [Title/Abstract]
#16	Moxa [Title/Abstract]
#17	Mugwort [Title/Abstract]
#18	#14 OR #15 OR #16 OR #17
#19	#13 And #18
